# Identification of structural brain alterations in adolescents with depressive symptomatology

**DOI:** 10.1016/j.brainresbull.2023.110723

**Published:** 2023-08-01

**Authors:** Johannah Bashford-Largo, R. James R. Blair, Karina S. Blair, Matthew Dobbertin, Ahria Dominguez, Melissa Hatch, Sahil Bajaj

**Affiliations:** a Multimodal Clinical Neuroimaging Laboratory (MCNL), Center for Neurobehavioral Research, Boys Town National Research Hospital, Boys Town, NE, USA; b Center for Brain, Biology, and Behavior, University of Nebraska-Lincoln, Lincoln, NE, USA; c Child and Adolescent Mental Health Centre, Mental Health Services, Capital Region of Denmark, Copenhagen, Denmark; d Child and Adolescent Inpatient Psychiatric Unit, Boys Town National Research Hospital, Boys Town, NE, USA; e The University of Texas MD Anderson Cancer Center, Houston, TX, USA

**Keywords:** Depression, Neuroimaging, Adolescents, Cortical volume

## Abstract

**Introduction:**

Depressive symptoms can emerge as early as childhood and may lead to adverse situations in adulthood. Studies have examined structural brain alternations in individuals with depressive symptoms, but findings remain inconclusive. Furthermore, previous studies have focused on adults or used a categorical approach to assess depression. The current study looks to identify grey matter volumes (GMV) that predict depressive symptomatology across a clinically concerning sample of adolescents.

**Methods:**

Structural MRI data were collected from 338 clinically concerning adolescents (mean age = 15.30 SD=2.07; mean IQ = 101.01 SD=12.43; 132 F). Depression symptoms were indexed via the Mood and Feelings Questionnaire (MFQ). Freesurfer was used to parcellate the brain into 68 cortical regions and 14 subcortical regions. GMV was extracted from all 82 brain areas. Multiple linear regression was used to look at the relationship between MFQ scores and region-specific GMV parameter. Follow up regressions were conducted to look at potential effects of psychiatric diagnoses and medication intake.

**Results:**

Our regression analysis produced a significant model (*R*^*2*^ = 0.446, *F*(86, 251) = 2.348, *p <* 0.001). Specifically, there was a negative association between GMV of the left parahippocampal (B = −0.203, p = 0.005), right rostral anterior cingulate (B = −0.162, p = 0.049), and right frontal pole (B = −0.147, p = 0.039) and a positive association between GMV of the left bank of the superior temporal sulcus (B = 0.173, p = 0.029). Follow up analyses produced results proximal to the main analysis.

**Conclusions:**

Altered regional brain volumes may serve as biomarkers for the development of depressive symptoms during adolescence. These findings suggest a homogeneity of altered cortical structures in adolescents with depressive symptoms.

## 1. Introduction

Major Depressive Disorder (MDD) is one of the most common mental health disorders in adults and adolescents (NIMH, 2021). Depression is associated with an ongoing depressive state and loss of interest in former enjoyable activities that has a large impact on one’s life (American Psychiatric Association, 2013). Depression is one of the top leading causes of disability worldwide and can become a serious mental health condition, sometimes tragically leading to suicide (WHO, 2021). Identifying symptoms of depression early on in childhood and/or during adolescence can help minimize risk factors, as adolescent depression can persist into adulthood (Naicker et al., 2013). Neuroimaging studies have shown both functional and structural brain differences in individuals with depression compared to those without. However, findings have been inconsistent and little previous work has taken a dimensional approach to studying the symptoms underlying depression.

With respect to structural neuroimaging findings, the most consistent results have implicated reduced cortical volumes within ventral and medial regions of frontal cortex including dorsomedial prefrontal cortex (dmPFC), orbitofrontal cortex (OFC) and (rostral) anterior cingulate cortex (ACC) (Enigma et al., 2017; Lacerda et al., 2004; Li et al., 2010; Rodríguez-Cano et al., 2014; Schmaal et al., 2020; Wang et al., 2017). In addition, the insular cortex has been generally shown to have reduced cortical thickness and volume, particularly in the left hemisphere (Enigma et al., 2017; Lai and Wu, 2014; Myoraku et al., 2022; Zhang et al., 2016; Zhao et al., 2017a; Zheng et al., 2021) – however this is not always the case (Tu et al., 2012; Wang et al., 2017; Zuo et al., 2018). Relative to healthy controls, MDD symptomology has been associated with a reduction in CV (Shen et al., 2021), cortical thickness (Enigma et al., 2017; Niu et al., 2017), and cortical surface area (Peng et al., 2015) in regions within the temporal cortex. However, the temporal pole specifically has shown to have both volumetric reductions (Rodríguez-Cano et al., 2014) and an increase in thickness (Fallucca et al., 2011; van Eijndhoven et al., 2013) in those with depression.

When looking at subcortical structures, the literature frequently points to the amygdala and hippocampus (Bashford-Largo et al., 2022; MacMaster et al., 2014; Myoraku et al., 2022; Roberson-Nay et al., 2006) and, to a certain extent, the thalamus (Ancelin et al., 2019; Nugent et al., 2013; Zhang et al., 2012) as being implicated in individuals with depression. While functional studies have relatively consistently identified atypical amygdala responses to emotional stimuli in individuals with depression (Perlman et al., 2012; Roberson-Nay et al., 2006; Yang et al., 2010), structural studies have reported increased (Zuo et al., 2018), decreased (Bora et al., 2012; Gray et al., 2020; Myoraku et al., 2022; Zhao et al., 2017b) or no significant differences (Koolschijn et al., 2009; Schmaal et al., 2020) in amygdala volume. In contrast, most studies have shown volumetric reductions of the hippocampus in individuals with depression relative to comparison participants (Arnone et al., 2013; MacMaster et al., 2014; Myoraku et al., 2022; Rodríguez-Cano et al., 2014; Schmaal et al., 2020; Zhao et al., 2017a) with a few exceptions showing increases in volume or no change (Han et al., 2014; Zuo et al., 2018). The thalamus has been reported to show both grey matter reduction (Ancelin et al., 2019; Hagan et al., 2015; Lu et al., 2016; Nugent et al., 2013) and increases (Wang et al., 2017; Zhang et al., 2012; Zhao et al., 2014) in patients with depression compared to healthy controls.

The inconsistent and diverse findings regarding depression-related structural brain alterations have left researchers with more questions than answers. This study aims to address the inconsistent and widespread alterations associated with depression by utilizing a dimensional approach to target depression symptomatology. Many morphometric studies looking at depression tend to focus on group comparison using individuals with depression vs healthy controls (ENIGMA et al., 2017; Suh et al., 2019; van Eijndhoven et al., 2016). As there are varying levels of symptom severity and heterogeneity among individuals, using depressive symptomatology instead of strict categorical diagnoses can lead to more generalizable results (Forbes et al., 2016; Salicru, 2020).

In sum, our primary goal was to study whole-brain region-specific GMV parameters in clinically concerning adolescents with depressive symptoms. Based on prior research, we hypothesized that there would be GMV decreases in the prefrontal cortex, ACC, insula, and hippocampus associated with depressive symptoms in adolescents.

## 2. Methods

### 2.1. Participants

Participants were recruited from a residential care facility in the Midwest and from the surrounding community. Participants were referred for various behavioral and mental health problems. The group of participants included 338 clinically concerning adolescents (had at least one of the seven clinical diagnoses determined by the staff psychiatrist) (132 F/206 M) with a mean age of 15.30 (SD = 2.07) and mean IQ of 101.01 (SD = 12.43); see Table 1 for full demographics on participants and diagnoses/medications. Exclusion criteria included braces, claustrophobia, active substance dependence, pervasive developmental disorders, Tourette’s syndrome, lifetime history of psychosis, neuro-logical disorders, head trauma, non-English speaking, and presence of active safety concerns. Clinical characterization was done through psychiatric interviews by licensed and board-certified child and adolescent psychiatrists with the participants and their parents to adhere closely to common clinical practice. All participants and their parents provided written informed assent/consent prior to enrollment. The study protocol was approved by the Institutional Review Board at the residential care facility.

## 3. Data collection

### 3.1. Neuroanatomical data

High resolution structural MRI (T1-weighted) data were collected using a 3-Tesla Siemens MRI scanner located at Boys Town National Research Hospital. Each participant was instructed to rest, relax, and try their best to minimize head movement during the entire scan. Whole-brain anatomical data for each participant were acquired using a 3D magnetization-prepared rapid acquisition gradient echo (MPRAGE) sequence, which consisted of 176 axial slices (slice thickness = 1 mm, voxel resolution = 0.9 × 0.9 × 1 mm^3^, repetition time = 2200 ms; echo time = 2.48 ms; matrix size = 256 ×208; field of view (FOV) = 230 mm, and flip angle = 8°).

### 3.2. General Intelligence (IQ)

The Wechsler Abbreviated Scale of Intelligence II (WASI-II) (Wechsler, 2011) was used to estimate IQ in the domains of perceptual reasoning, verbal comprehension, and Full-Scale IQ (FSIQ). FSIQ scores have high reliability (*α* = 0.98) and strong correlations (*r* = 0.92) with scores on the full Wechsler Adult Intelligence Scale (WAIS)-III (Wechsler, 1999, 1997) and were used in the current context.

#### 3.2.1. MFQ

*The Mood and Feelings Questionnaire* (MFQ, (Costello and Angold, 1988) is a self-report questionnaire that looks at depressive symptoms in youth and young adults. The MFQ uses a 3 point scale (0 = not true, 1 = sometimes true, 2 = true) with a suggested cutoff of 28 – 29 suggesting the presence of depression (score range of 0 – 60) (Thabrew et al., 2018; Daviss, 2006). Higher scores indicate higher presence of current depressive symptomatology. The MFQ has shown to be useful in both community and clinical samples ((Daviss et al., 2006) as well as the use of discriminating between depressed and non-depressed samples (Kent et al., 1997). The MFQ has been shown to have high criterion validity (Rhew et al., 2010) and excellent internal consistency (α = 0.91–0.93) (Thabrew et al., 2018).

## 4. Image preprocessing

The “recon-all” pipeline from the FreeSurfer toolbox (Version 6.0; https://surfer.nmr.mgh.harvard.edu) was used to process the anatomical brain images (Dale et al., 1999; Fischl et al., 1999) and for esti-mating morphometry parameters. All preprocessing steps to ensure overall accuracy were performed in FreeSurfer. Scans were corrected for motion and normalized, and the skull was removed via Freesurfer’s algorithm (Segonne et al., 2005). Quality control measures follow the ENIGMA cortical surface segmentations protocol (see www.enigma.ini.usc.edu for full details and scripts). Subjects were inspected for possible outliers, and if outliers were present, detailed checks for segmentation issues were examined before proceeding. MATLAB scripts (“MATLAB, ”, 2021) were run for checking internal and external areas to check labeling and correct rendering of surfaces. Subjects are rated as “pass” (no issues), “moderate” (certain regions might not pass), or “fail” (severe issues). We obtained 616 participants who passed our quality control and 338 were chosen for this study who had complete assessment data and matched status for clinically concerning.

## 5. Data extraction and preparation

The whole brain was parcellated into 68 cortical regions using the Desikan-Killiany atlas (Desikan et al., 2006) and 14 subcortical regions using whole-brain default automated segmentation (Fischl et al., 2002) using FreeSurfer. Subject-wise and hemispheric-wise measures of cortical and subcortical volume (CV/SCV) were estimated from the 68 cortical and 14 subcortical areas respectively. The estimated intracranial volume (ICV) represents the overall head size. The *mri_surf2surf*, *mri- s_anatomical_stats*, and *aparcstats2table* (B. Fischl et al., 1999) Freesurfer pipelines were used to extract CV/SCV and ICV measures.

## 6. Data analysis

A multiple linear regression to predict depressive scores via the MFQ was run with the 68 cortical and 14 subcortical volumes as independent variables. As sex and ICV were significantly correlated with MFQ scores (r = −281, p < 0.001 and r = −0.131, p = 0.016 respectively) they were included in the model as covariates. Multiple participants were clinically diagnosed with various internalizing and externalizing diagnoses. Of the seven psychiatric diagnoses the clinical youth had, generalized anxiety disorder and post-traumatic stress disorder correlated significantly with MFQ scores (both p < 0.001), thus we added these diagnoses to the model as covariates as well.

## 7. Follow-up analyses

As the study involved participants with psychiatric diagnoses, there were 122 adolescents taking medications at the time of the study; see Table 1. Stimulants and SSRIs significantly correlated with MFQ scores (r = 0.128, p = 0.018 and r = 0.107, p = 0.049 respectively). Given this potential confound, the analysis was re-run with these medications included in the overall model (scored 1 for “yes” or 0 for “no”).

## 8. Results

Our regression analysis produced a significant model (*R*^*2*^ = 0.446, *F* (86, 251) = 2.348, *p <* 0.001). Specifically, there was a negative association between GMV of the left parahippocampal (B = ‒0.203, p = 0.005), right rostral ACC (B = ‒0.162, p = 0.049), and right frontal pole (B = ‒0.147, p = 0.039), suggesting that smaller volume in this area is associated with higher MFQ scores. There was a positive association between GMV of the left banks of the superior temporal sulcus (B = 0.173, p = 0.029), suggesting that larger volumes in this area is associated with higher MFQ scores.

## 9. Follow up analysis

Results were proximal to the main analysis and produced a significant model (*R*^*2*^ = 0.451, *F*(88, 249) = 2.321, *p <* 0.001). There was still a negative association between GMV of the left parahippocampal (B = −0.208, p = 0.004), right rostral ACC (B = −0.174, p = 0.036), and right frontal pole (B = −0.141, p = 0.048), suggesting that smaller volumes in this area is associated with higher MFQ scores. There was also still a positive association between GMV of the left banks of the superior temporal sulcus (B = 0.166, p = 0.035) and MFQ scores.

## 10. Discussion

The goal of this study was to identify alteration in region-specific GMV that predicted depressive scores in adolescents. Consistent with our hypothesis, we saw decreases in prefrontal regions (frontal pole) and anterior cingulate cortex (ACC) that contributed to the prediction of MFQ scores. There was one region, the banks of the superior temporal sulcus, that had a positive association with depressive symptomology. We did not see significance within any subcortical volumes.

The current study expands on prior work that explores structural disruptions in depression symptomatology. The results have been heterogeneous, and these results help fill in the unreliable findings surrounding the neurobiology of depression. Despite these differences, multiple functional (Diener et al., 2012; Holt et al., 2016; Johnstone et al., 2007) and structural (Enigma et al., 2017; Lacerda et al., 2004; Li et al., 2010; Rodríguez-Cano et al., 2014; Schmaal et al., 2020; Wang et al., 2017) studies have implicated regions of frontal cortex in the pathology of depression. The frontal lobe has many functions, many of which have been shown to be atypical in individuals with depression; e. g., working memory, awareness of self, and decision making (Park et al., 2019; Schiller et al., 2013; Shad et al., 2011).

Consistent with prior studies, we found a negative association in volume in the right ACC and right frontal pole (Webb et al., 2014) to depression symptoms. The ACC has been shown to be implicated in emotion and mood regulation, and its reduction in individuals with MDD is one of the most consistent findings in MDD literature (Bora et al., 2012; Du et al., 2012). The frontal pole plays an important role in higher order social and emotional processes, such as attending to one’s own emotional states (Gilbert et al., 2006; Raju et al., 2021). Multiple studies have seen cortical volume reductions in the frontal pole/medial prefrontal areas in individuals with depression (Bludau et al., 2016; Grieve et al., 2013; Koolschijn et al., 2009).

There have been mixed reports of structural (Caetano et al., 2004; Takahashi et al., 2010) and functional (Hamilton et al., 2012; Weng et al., 2019) alterations in superior temporal regions seen in conjunction with depression. The superior temporal gyrus has been shown to be implicated in emotional processing (Gallagher and Frith, 2003; Morawetz et al., 2015). Our findings of increased GMV within the banks of the superior temporal sulcus add to previous atypical results seen in temporal areas, which could explain some of the emotional dysfunction commonly seen in depression. Also within the temporal cortex is the parahippocampal gyrus which is part of the paralimbic cortex and has been shown to play a role in emotional and contextual processing (Kumfor et al., 2018; van Eijndhoven et al., 2013). Prior studies have also seen decreased volumes within the parahippocampal gyrus in those with depressive symptoms (Peng et al., 2011; Rodríguez-Cano et al., 2014; Zheng et al., 2021). The parahippocampal gyrus is part of the DMN network, which is commonly seen to have disruptions within internalizing disorders such as anxiety and depression (Bashford-Largo et al., 2022; Zeng et al., 2012).

With respect to GMV of subcortical areas and depression, the literature has shown mixed results. While the hippocampus frequently shows cortical reductions, the hippocampus and the amygdala have been shown to have increased (Han et al., 2014; Zuo et al., 2018), decreased (Bora et al., 2012; MacMaster et al., 2014) or no differences (Koolschijn et al., 2009; Zuo et al., 2018) in individuals with MDD compared to healthy controls. In this study, we did not have any significant findings of altered GMV in the hippocampus or amygdala. However, studies have shown volumetric differences in frontal-subcortical areas in various psychiatric disorders including those with depression (Kong et al., 2014). Alterations in these frontal-subcortical circuits have been shown to influence cognitive and emotional processing (Drevets, 2001; Marchand, 2010), which have been shown to have deficits in individuals with depression (Joormann and Gotlib, 2010; Joormann and Quinn, 2014; Park et al., 2019). In this study, we found cortical alterations in frontal areas, possibly giving reason to disruptions in these frontal-subcortical circuits.

Our study sheds light on morphometric differences seen on the depression continuum. Our findings are consistent with prior studies showing negative associations with frontal volumes (Bora et al., 2012; Du et al., 2012; Koolschijn et al., 2009; Webb et al., 2014), and provide more insight to possible morphometric associations of temporal regions and depression. One of the unique aspects of this present study is the adolescent age range, which is an age group looked at much less than adults in depressive studies. Age of onset has been shown to help further demystify the heterogeneity of psychiatric disorders. For example, those with early adult age onset depression will tend to have higher comor-bidities of personality disorders and higher levels of neuroticism (Bukh et al., 2011). Another distinct feature of this study is the use of depressive symptomatology instead of depression diagnosis, such as Major Depressive Disorder, as analysis based on diagnoses can be restrictive due to the variability in diagnostic criteria. Assessing structural alterations via symptoms can provide a more representative analysis of those with depression. To our knowledge, this is one of the few studies to take such dimensional approach to study a large sample of adolescents with a range of depressive symptomatology while utilizing a whole-brain region-specific structural analysis.

There are a few limitations to this study. First, depression symptomatology is highly comorbid with other diagnoses (Steffen et al., 2020). This is seen in our sample as well (Table 1). Because of this high comorbidity, it could be said that one of these comorbid diagnoses could be contributing to the results. To address this concern, we identified internalizing diagnoses that significantly correlated with MFQ scores in our sample and added them as covariates and found results proximal to the main analysis. Second, several of our adolescents were on medications at the time of the study. Therefore, we ran a follow up multiple linear regression with stimulants and SSRIs as covariates and found results proximal to the main analysis. Third, there was a degree of over- sampling of male participants. While sex was included as a covariate in the statistical model, it remains possible that some of the findings are sex-specific and may not generalize to female participants.

**Fig. 1.**
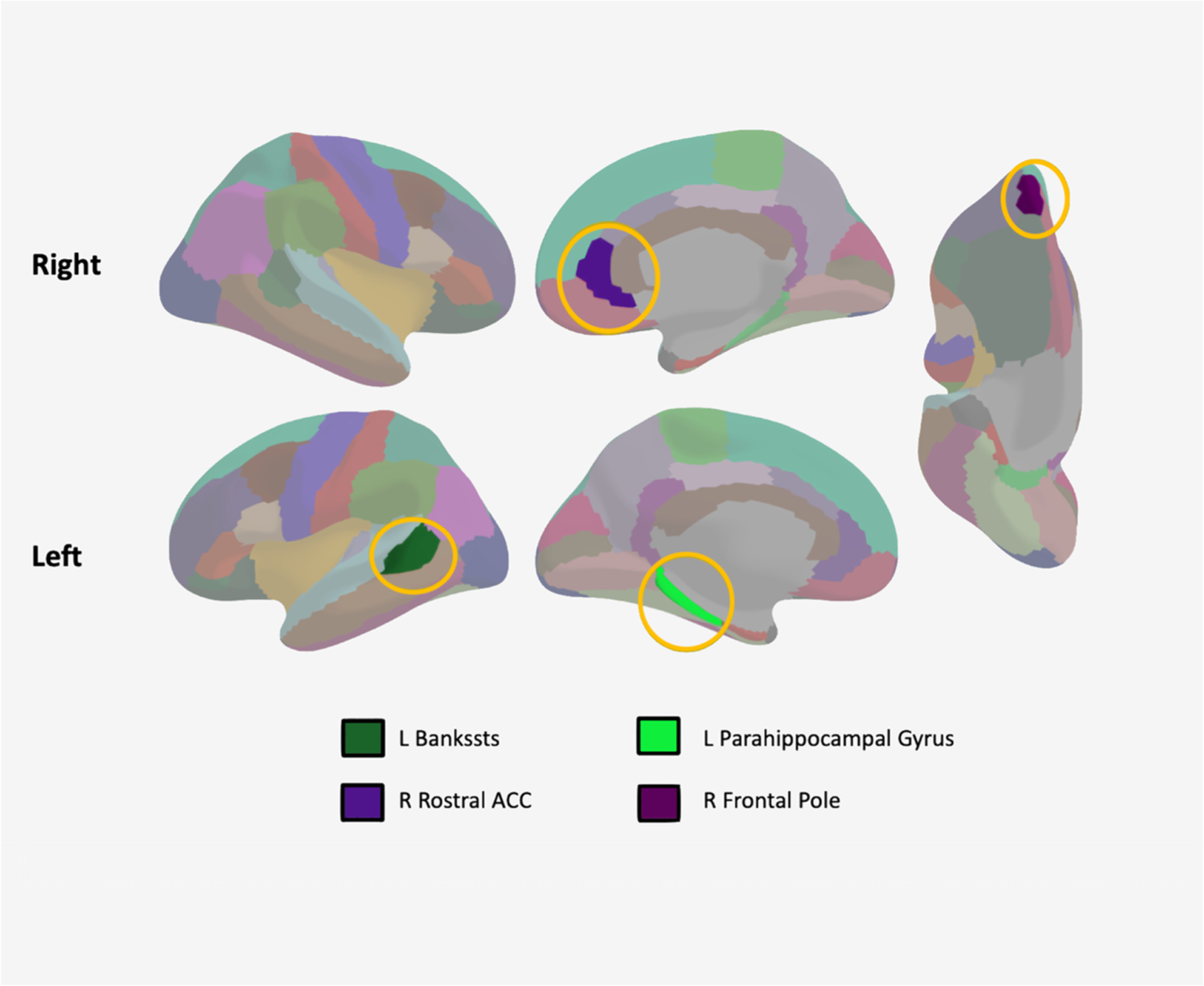
Regions with Significant Cortical Volumes in Model: Regions showing significant associations with MFQ scores and their cortical volumes including the left banks of the temporal sulcus (bankssts), left parahippocampal gyrus, right anterior cingulate cortex (ACC), and right frontal pole. All showed negative associations except for the left bankssts, which showed a positive association with MFQ scores Key to Figure: L:left, R:right, Bankssts: banks of the temporal sulcus, ACC: Anterior Cingulate Cortex.

In conclusion, our study revealed volumetric alteration in specific brain regions that predicted depressive symptomatology in a clinically concerning sample of adolescents. Depression is an extremely hetero- geneous diagnosis which makes classification difficult in therapeutic settings (Fried, 2017). However, the current data suggest some more general neurobiological risk markers for those with higher level depressive symptoms. The identification of specific markers that are unique to depression symptomatology can have large clinical implications and help aid in early intervention strategies.(Fig. 1).

## Figures and Tables

**Table 1:** Demographics Characteristics.

Characteristics	Sample (N = 338)
**Sex**	132 Female/206 Male
**Age**	15.30 (SD = 2.07)
**IQ**	101. 01 (SD = 12.43)
**ICV (x10**^**6**^**) in mm**^**3**^	1.48 (SD = 1.47)
**MFQ Score**	12.52 (SD = 11.98), possible range 0–60, sample range
***Clinical Diagnoses***	0–60
**MDD**	99 (29.3%)
**GAD**	99 (29.3%)
**SAD**	108 (32%)
**PTSD**	45 (13.3%)
**CD**	168 (49.7%)
**ADHD**	232 (68.6%)
**ODD**	215 (63.6%)
**At least one diagnosis**	320 (94.67%)
***Medications* Antipsychotic**	26 (7.7%)
**SSRIs**	58 (17.2%)
**Stimulants**	76 (22.5%)

Key to table. MDD=Major Depressive Disorder; IQ=Intelligent Quotient; ICV=Intercranial Cortical Volume; MFQ=Mood and Feelings Questionnaire; MDD=Major Depressive Disorder; GAD=Generalized Anxiety Disorder; SAD=Social Anxiety Disorder; PTSD=Post Traumatic Stress Disorder; CD=Conduct Disorder; ADHD=Attention Deficit Hyperactivity Disorder; ODD= Oppositional Defiant Disorder; SSRIs=Selective Serotonin Reuptake Inhibitors; SD=Standard Deviation

## Data Availability

Data will be made available on request. The data that support the findings of this study are available from the corresponding author upon reasonable request. The data are not publicly available due to IRB restrictions.
